# Epigenetic Regulation of Uterine Smooth Muscle Tumors: Histone Modifications in Uterine Fibroids and Leiomyosarcoma

**DOI:** 10.3390/biology15110838

**Published:** 2026-05-27

**Authors:** Qiwei Yang

**Affiliations:** Department of Obstetrics and Gynecology, University of Chicago, Chicago, IL 60637, USA; yangq@bsd.uchicago.edu

**Keywords:** uterine smooth muscle tumors, uterine fibroids, uterine leiomyosarcoma, epigenetic regulation, histone modification, histone acetylation, histone methylation, chromatin remodeling, DNA methylation, non-coding RNA, histone-modifying enzymes, epigenetic biomarkers, epigenetic therapy, tumor progression

## Abstract

Uterine smooth muscle tumors (USMTs) represent a diverse group of neoplasms arising from the myometrium, ranging from benign uterine fibroids (leiomyomas) to highly aggressive uterine leiomyosarcoma. While genetic alterations contribute to tumor development, growing evidence highlights the crucial role of epigenetic regulation in shaping tumor behavior. Among these mechanisms, histone modification has emerged as a key regulator of chromatin structure and gene expression. Histone modifications, including acetylation, methylation, phosphorylation, ubiquitination, ADP-ribosylation, and SUMOylation, are dynamically controlled by epigenetic regulators known as writers, erasers, and readers, which collectively modulate transcriptional programs involved in cell proliferation, differentiation, and stress responses. Recent studies indicate that dysregulation of histone-modifying enzymes contributes to the pathogenesis of USMTs by altering chromatin accessibility and transcriptional networks. In uterine fibroids, histone modifications are associated with hormone-responsive signaling pathways, extracellular matrix deposition, and abnormal smooth muscle cell proliferation. In contrast, uterine leiomyosarcoma exhibits extensive epigenetic reprogramming characterized by aberrant histone acetylation and methylation patterns, dysregulated chromatin regulators, and activation of oncogenic signaling pathways that promote tumor aggressiveness and genomic instability. Importantly, histone modifications interact with other epigenetic mechanisms, including DNA methylation, non-coding RNA–mediated regulation, and RNA epitranscriptomics, forming complex networks that influence tumor initiation and progression. This narrative review summarizes current knowledge on histone modification pathways and their roles in USMT biology, highlighting the functions of histone-modifying enzymes, their interactions with other epigenetic mechanisms, and their impact on tumor development. In addition, this review discusses emerging therapeutic strategies targeting epigenetic regulators, including inhibitors of histone deacetylases, histone methyltransferases, and readers, as well as potential epigenetic biomarkers for diagnosis and prognosis. Finally, this review outlines future research directions, including multi-omics integration, and advanced epigenomic technologies, which may provide deeper insights into the epigenetic landscape of USMTs and facilitate the development of personalized therapeutic approaches.

## Simple Summary

Uterine smooth muscle tumors are neoplasms arising from the smooth muscle layer of the uterus. These tumors range from common non-cancerous uterine fibroids to rare but aggressive cancers. Although changes in genes play a role in how these tumors form, recent research shows that another layer of control, called epigenetic regulation, is also very important. This study focuses on changes in proteins that help package DNA, which can turn genes on or off without altering the DNA sequence itself. When these processes are disrupted, they can lead to uncontrolled cell growth, increased tissue buildup, and tumor progression. In uterine fibroids, these changes are linked to hormone responses and excessive tissue formation, while in more aggressive tumors, such as uterine leiomyosarcoma, they contribute to rapid growth and instability of genetic material. This review also highlights how these changes interact with other regulatory systems in the cell to influence disease development. Importantly, targeting these processes may offer new treatment options beyond traditional therapies. Understanding these mechanisms may help improve diagnosis, guide treatment decisions, and lead to more personalized approaches for patients with uterine tumors.

## 1. Introduction

### 1.1. Uterine Smooth Muscle Tumors

Uterine smooth muscle tumors (USMTs) represent a heterogeneous spectrum of neoplasms arising from the smooth muscle cells of the myometrium, the muscular layer of the uterus. These tumors range from common benign lesions to rare but highly aggressive malignancies and are primarily classified into benign uterine fibroids (UFs), also known as uterine leiomyomas, and malignant uterine leiomyosarcoma (uLMS) [1,2]. UFs are typically well-circumscribed, hormone-responsive tumors associated with abnormal uterine bleeding, pelvic pressure, pain, and infertility [3], whereas uLMS is characterized by rapid growth, early metastatic potential, frequent recurrence, and poor prognosis despite surgical intervention and adjuvant therapy [2,4,5]. At the molecular level, these tumors also display distinct genetic and epigenetic alterations, underscoring divergent pathogenic mechanisms despite their shared tissue of origin [2,4]. Although both tumor types originate from uterine smooth muscle cells, they differ substantially in their biological behavior, clinical outcomes, and molecular characteristics [2,6,7].

#### 1.1.1. Uterine Fibroids

UFs are the most common benign tumors of the female reproductive tract and affect a large proportion of women of reproductive age [8,9]. These tumors are typically well-circumscribed and composed of smooth muscle cells and abundant extracellular matrix (ECM) components such as collagen and fibronectin [10,11]. UF growth is strongly influenced by ovarian steroid hormones, particularly estrogen and progesterone, and they frequently exhibit dysregulated signaling pathways related to cell proliferation, ECM deposition, and hormonal responsiveness [12,13,14]. Molecular studies have identified recurrent genetic alterations in UFs, including mutations in genes such as *MED12*, rearrangements involving *HMGA2*, and alterations in fumarate hydratase in a subset of hereditary cases [6,15,16]. Genome-wide association studies (GWAS) have identified multiple genetic variants associated with gynecological disorders, yet their functional mechanisms remain largely unresolved. In UFs, risk loci involving sex hormone-binding globulin levels, obesity-related modifiers, and genes such as PRMT6 have been linked to disease susceptibility, along with shared genetic signals for hypertension and reproductive traits [17,18,19,20]. Although UFs are benign and generally slow growing, their high prevalence and associated morbidity make them a major health concern worldwide [21].

#### 1.1.2. Uterine Leiomyosarcoma

In contrast, uLMS is a rare but highly aggressive malignant tumor arising from uterine smooth muscle cells [2,22]. Unlike UFs, which are typically well-defined and slow growing, LMS are characterized by rapid growth, cellular atypia, high mitotic activity, and a strong propensity for recurrence and metastasis [1,23]. Patients with uLMS often present with nonspecific symptoms such as abnormal uterine bleeding, pelvic pain, or rapidly enlarging uterine masses, making early diagnosis challenging [2,5]. Importantly, uLMS is generally considered to arise de novo rather than through malignant transformation of preexisting UFs [1]. Molecularly, these tumors display complex genomic alterations, including frequent mutations or loss of tumor suppressor genes such as TP53, RB1, and PTEN, along with widespread chromosomal instability [7]. These alterations contribute to uncontrolled proliferation, genomic instability, and aggressive tumor behavior.

Despite their distinct clinical and pathological characteristics, benign UFs and malignant uLMS share a common cellular origin in uterine smooth muscle cells, highlighting the importance of understanding the molecular mechanisms that regulate smooth muscle cell growth and differentiation in the uterus [22]. Increasing evidence suggests that dysregulation of signaling pathways, epigenetic mechanisms, and tumor microenvironment interactions plays important roles in the development and progression of USMTs [3,9]. Improved understanding of these mechanisms may facilitate the development of better diagnostic markers and therapeutic strategies for both benign and malignant forms of these tumors.

### 1.2. Histone Modifications

Histone modifications represent a principal layer of epigenetic regulation. In eukaryotic chromatin, DNA is packaged into nucleosomes composed of histone octamers containing histones H2A, H2B, H3, and H4. These nucleosomes serve as substrates for a wide range of post-translational modifications (PTMs) that occur predominantly on the N-terminal tails of histones. Such modifications alter chromatin accessibility, nucleosome stability, and the recruitment of regulatory protein complexes, thereby influencing transcriptional activity and genome organization [24,25]. Histone PTMs function through a coordinated system of three classes of regulators: writers, which deposit modifications; erasers, which remove them; and readers, which recognize and interpret these marks to mediate downstream chromatin functions.

#### 1.2.1. Writers That Deposit Histone Modifications

Histone modifications are deposited by a diverse group of enzymes collectively referred to as histone modification writers, which catalyze the addition of specific chemical groups to histone proteins and thereby regulate chromatin structure and gene expression [25]. These enzymes install a broad spectrum of covalent marks, including acetylation, methylation, phosphorylation, ubiquitination, and other modifications, primarily on the N-terminal tails of histones H3 and H4 [26]. Through these activities, histone writers influence chromatin accessibility and facilitate the recruitment of regulatory complexes involved in transcription, DNA replication, and DNA repair [16,27].

Among the most extensively studied histone writers are histone acetyltransferases (HATs), which catalyze the transfer of acetyl groups from acetyl-CoA to lysine residues on histones in a process known as histone acetylation [24,28]. Acetylation neutralizes the positive charge of lysine residues, weakening histone–DNA interactions and promoting a more open chromatin configuration associated with transcriptional activation. Major HAT families include the GNAT family, represented by KAT2A and KAT2B, which acetylate histone H3 at residues such as H3K9 and H3K14 [29]. The MYST family, including TIP60 and MOF, contributes to acetylation of histone H4, particularly at H4K16 [30]. In addition, the transcriptional coactivators EP300 and CREBBP play central roles in enhancer activation and transcriptional regulation by depositing marks such as H3K27ac [31].

Another major group of histone writers consists of histone methyltransferases (HMTs), which catalyze the transfer of methyl groups from S-adenosylmethionine to lysine or arginine residues on histones in a process referred to as Histone Methylation. Histone methylation can occur as mono-, di-, or trimethylation and may correlate with either transcriptional activation or repression depending on the modified residue [32,33]. For example, methylation of histone H3 at lysine 4 (H3K4) is typically associated with active transcription and is catalyzed by members of the MLL/SET1 family such as KMT2A [34]. In contrast, the repressive mark H3K27me3 is deposited by the Polycomb methyltransferase EZH2, a catalytic component of the PRC2 complex that mediates gene silencing during development and disease [35]. Additional HMTs such as SETD2 catalyze H3K36 methylation linked to transcription elongation [36], whereas enzymes including SUV39H1 mediate H3K9 methylation associated with heterochromatin formation [37].

In addition to acetylation and methylation, several other classes of enzyme’s function as histone writers. Histone kinases catalyze the addition of phosphate groups to serine, threonine, or tyrosine residues in a process known as histone phosphorylation, which often occurs in response to cellular stress or during cell-cycle progression [38,39]. For instance, AURKB (Aurora B kinase) phosphorylates histone H3 at serine 10 (H3S10ph), a modification associated with chromosome condensation during mitosis [40]. In response to DNA damage, kinases such as ATM and ATR phosphorylate histone variant H2AX to generate γH2AX, which functions as a key signal for the recruitment of DNA repair factors [41,42,43].

Another important class of histone writers includes histone ubiquitin ligases, which catalyze the covalent attachment of ubiquitin molecules to histone proteins in a process termed histone ubiquitination. Unlike polyubiquitination that targets proteins for proteasomal degradation, histone ubiquitination often serves regulatory roles in chromatin [44]. A well-characterized example is monoubiquitination of histone H2B at lysine 120 (H2BK120ub), catalyzed by the E3 ligase complex containing RNF20 and RNF40, which promote transcription elongation and facilitates downstream histone methylation events such as H3K4 and H3K79 methylation [45]. Similarly, the Polycomb repressive complex component RING1B catalyzes monoubiquitination of H2A at lysine 119 (H2AK119ub), contributing to transcriptional repression and stable gene silencing [46,47].

ADP-ribosylation is a reversible post-translational histone modification in which one or more ADP-ribose units are transferred from NAD^+^ to specific amino acid residues on histone proteins, most commonly glutamate, aspartate, or lysine. This reaction is primarily catalyzed by poly(ADP-ribose) polymerases (PARPs), particularly PARP1 and PARP2, which are rapidly activated in response to DNA damage and chromatin stress. ADP-ribosylation can occur as mono-ADP-ribosylation or as poly(ADP-ribosyl)ation, leading to the formation of branched polymer chains that alter chromatin structure. Functionally, this modification generally promotes chromatin relaxation, thereby facilitating DNA repair processes such as base excision repair and recruitment of DNA repair factors. In addition to its role in genome stability, ADP-ribosylation also influences transcriptional regulation by modulating nucleosome dynamics and histone–DNA interactions, and it is tightly regulated by ADP-ribosyl hydrolases such as PARG, which remove ADP-ribose chains to restore chromatin homeostasis [48].

In addition to these canonical modifications, other histone-modifying enzymes further diversify chromatin regulation. One such modification is SUMOylation, in which small ubiquitin-like modifier (SUMO) proteins are covalently attached to histones by SUMO-conjugating enzymes, primarily UBC9 [49]. Histone SUMOylation is generally associated with transcriptional repression and chromatin compaction, as it facilitates recruitment of transcriptional corepressors and histone deacetylase complexes [50]. Additionally, histones can undergo ADP-ribosylation mediated by enzymes such as PARP1, particularly during DNA damage responses, where this modification promotes chromatin relaxation and recruitment of DNA repair factors [51,52].

**Figure 1:**
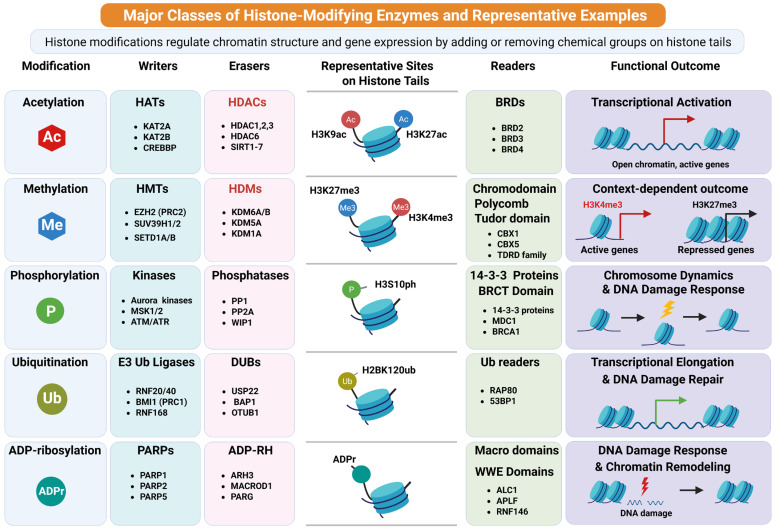
Major classes of histone-modifying enzymes and representative examples. Schematic overview of the principal histone modifications and the major classes of enzymes involved in the establishment, removal, and recognition of these epigenetic marks. Histone “writers” catalyze the addition of chemical modifications, whereas “erasers” remove these marks, and “readers” recognize modified histones to mediate downstream chromatin-associated functions. Representative modifications include histone acetylation, methylation, phosphorylation, ubiquitination, and ADP-ribosylation, together with their associated regulatory enzymes and representative histone residues. The figure also summarizes the major biological consequences of these modifications, including transcriptional activation or repression, chromatin remodeling, DNA damage response, chromosome dynamics, and regulation of gene expression programs. Dysregulation of these histone-modifying pathways contributes to the pathogenesis of UFs and uLMS through altered chromatin states and ab errant transcriptional control. Abbreviations: Ac; histone acetyltransferase; Me: methylation; P: phosphorylation; Ub: ubiquitination; ADP-r: ADP-ribosylation; HAT: histone acetyltransferase; HMT: histone methyltransferase; HK: histone kinase; HDAC: histone deacetylase; PARP: ADP-ribosyltransferase; HDM; histone demethylase; HP: histone phosphatase; DUB: deubiquitinase, ADP-RH: ADP-ribosylhydrolases.

Collectively, these diverse classes of histone modification writers establish a complex and dynamic network of chromatin marks that regulate genome function. By depositing specific PTMs on histone tails, these enzymes contribute to the formation of the histone code, a multilayered regulatory system that integrates environmental signals and developmental cues to control chromatin organization and gene expression (Figure 1).

#### 1.2.2. Erasers That Remove Histone Modifications

In contrast to histone writers that deposit covalent marks, histone modification erasers remove these modifications and thereby restore or reset chromatin states [24,53]. These enzymes play an essential role in maintaining the dynamic nature of chromatin by reversing histone modifications in response to developmental cues, environmental signals, and cellular stress [25,54]. Through the removal of specific marks, histone erasers regulate chromatin accessibility, transcriptional activity, and genome stability, forming a critical component of epigenetic regulation [55,56].

One major class of histone erasers is histone deacetylases (HDACs), which catalyze the removal of acetyl groups from lysine residues on histones, thereby reversing Histone Acetylation [55]. Deacetylation restores the positive charge on lysine residues, strengthening histone–DNA interactions and promoting chromatin condensation, which is typically associated with transcriptional repression [57]. HDACs are grouped into several classes based on sequence homology and cofactor requirements. Class I HDACs, including HDAC1, HDAC2, and HDAC3, are primarily nuclear and regulate transcriptional repression in multiprotein complexes [57,58]. Class II HDACs, such as HDAC4 and HDAC5, shuttle between the nucleus and cytoplasm and participate in tissue-specific gene regulation [59]. In addition, the NAD^+^-dependent class III deacetylases, known as Sirtuins, including SIRT1, contribute to chromatin regulation and cellular metabolic responses [60,61].

Another important group of histone erasers is histone demethylases, which remove methyl groups from lysine or arginine residues and thereby reverse histone methylation. Histone demethylases are primarily divided into two families based on their catalytic mechanisms. The first family includes the flavin-dependent amine oxidase LSD1 (also known as KDM1A), which demethylates mono- and dimethylated H3K4 and H3K9 residues and plays an important role in transcriptional regulation [53,62]. The second and larger family consists of Jumonji C (JmjC) domain–containing demethylases, which use Fe(II) and α-ketoglutarate as cofactors to remove methyl groups from histone lysines [63]. Members of this family include KDM4A, which demethylates H3K9me3 and H3K36me3, and KDM6A and KDM6B, which remove the repressive H3K27me3 mark deposited by EZH2 [64,65]. These demethylases contribute to transcriptional activation and chromatin remodeling during development and disease [54,66].

Additional eraser enzymes regulate other types of histone modifications. For instance, histone deubiquitinases (DUBs) remove ubiquitin moieties from histones, thereby reversing histone ubiquitination. Deubiquitinases such as USP22 remove ubiquitin from histone H2B, influencing transcriptional elongation and chromatin remodeling [67]. Similarly, enzymes such as BAP1 deubiquitinate H2A, counteracting Polycomb-mediated gene repression [68,69]. Histone phosphorylation marks can also be reversed by histone phosphatases, including PP1 and PP2A, which remove phosphate groups from histones and contribute to the regulation of mitosis and DNA damage signaling [38,70].

Other specialized erasers regulate less common histone modifications. For example, SUMO-specific proteases (SENPs), such as SENP1, remove SUMO groups from histones and reverse SUMOylation, thereby modulating transcriptional repression and chromatin organization [71]. Additionally, ADP-ribosylation marks deposited during DNA damage responses can be removed by enzymes such as PARG, which hydrolyze ADP-ribose polymers and help restore chromatin structure following repair [72,73,74].

Collectively, histone modification erasers work in concert with writers and chromatin-binding readers to maintain the dynamic equilibrium of histone marks. By removing specific modifications, these enzymes allow chromatin states to be rapidly altered in response to cellular signals, ensuring precise control of gene expression, DNA repair, and genome stability (Figure 1).

#### 1.2.3. Readers (e.g., Bromodomain and Chromodomain Proteins) That Interpret These Marks

In addition to writers and erasers, the biological functions of histone modifications are executed through histone modification readers, which recognize and bind to specific histone marks and translate them into downstream chromatin-based processes [75,76]. These reader proteins contain specialized structural domains that selectively interact with particular post-translational modifications (PTMs) on histone tails. By recognizing these marks, histone readers recruit transcriptional regulators, chromatin remodeling complexes, and other effector proteins, thereby linking histone modifications to functional outcomes in gene expression, DNA repair, and chromatin organization [25,77,78,79,80]. Together with writers and erasers, histone readers contribute to the dynamic interpretation of the histone code, a key component of epigenetic regulation.

One of the best-characterized classes of histone reader domains is the bromodomain, which specifically recognizes acetylated lysine residues generated through histone acetylation [81,82]. Bromodomain-containing proteins are often associated with transcriptional activation because they bind to acetylated histones at active promoters and enhancers [83]. Members of the bromodomain and extra-terminal (BET) protein family, such as BRD4, play key roles in transcriptional regulation by recruiting transcriptional machinery and elongation factors to acetylated chromatin [84]. Through these interactions, bromodomain proteins help stabilize open chromatin configurations and promote gene activation [85,86].

Another important class of histone readers is chromodomain-containing proteins, which recognize methylated lysine residues produced by histone methylation [87,88,89]. Chromodomain proteins often participate in transcriptional repression and heterochromatin formation. For example, CBX5 (HP1α) binds to H3K9me3, a hallmark of heterochromatin, and contributes to chromatin compaction and gene silencing [87,88]. Similarly, Polycomb group proteins containing chromodomains recognize repressive marks such as H3K27me3 and facilitate stable transcriptional repression during development and differentiation [47,90,91].

Beyond bromodomains and chromodomains, several additional reader domains contribute to the interpretation of histone modifications. For instance, plant homeodomain (PHD) fingers recognize specific methylated histone residues, particularly H3K4me3, a mark associated with active promoters. Proteins such as TAF3 utilize PHD fingers to bind this modification and facilitate transcription initiation [92,93]. Other reader modules include Tudor domains, WD40 repeats, and MBT domains, which bind distinct histone methylation states and participate in diverse chromatin regulatory pathways [94,95]. Through these specialized domains, histone reader proteins integrate multiple histone marks and coordinate the recruitment of transcription factors, chromatin remodelers, and other regulatory complexes [75,96].

Collectively, histone reader proteins provide a molecular interface that translates histone modifications into biological outcomes. By selectively recognizing acetylated, methylated, phosphorylated, or ubiquitinated histone residues, these proteins ensure that epigenetic signals deposited by writers and removed by erasers are properly interpreted. This coordinated system enables cells to dynamically regulate chromatin structure and gene expression in response to developmental signals and environmental stimuli (Figure 1).

### 1.3. Rationale for Reviewing Histone Modifications in USMTs

Recent studies suggest that aberrant chromatin regulation may contribute to both benign and malignant uterine smooth muscle tumor biology. In UFs, alterations in chromatin remodeling and transcriptional regulation have been associated with recurrent mutations in genes such as *MED12*, which influence transcriptional machinery and chromatin architecture. Similarly, uLMS exhibits widespread epigenetic dysregulation alongside complex genomic alterations, including mutations in key tumor suppressor genes such as TP53 and RB1. These molecular changes are often accompanied by altered activity of histone-modifying enzymes and chromatin regulatory complexes, suggesting that epigenetic reprogramming may play an important role in tumor progression, cellular plasticity, and therapeutic resistance.

Given the dynamic and reversible nature of histone modifications, they also represent attractive targets for therapeutic intervention. Epigenetic therapies targeting histone-modifying enzymes, such as histone deacetylase inhibitors and methyltransferase inhibitors, have shown promise in several cancers, raising the possibility that similar approaches may be applicable to uterine tumors. A comprehensive review of histone modification pathways in these tumors is therefore timely and important. By summarizing current knowledge of histone modification writers, erasers, and readers in the context of UFs and uLMS, this narrative review aims to highlight emerging epigenetic mechanisms that may contribute to tumor pathogenesis and identify potential avenues for improved diagnostics and targeted therapies.

### 1.4. Knowledge Gaps and Purpose of the Review

Despite growing recognition of the importance of chromatin regulation in cancer biology, the role of histone modifications in USMTs remains relatively underexplored. Many studies have focused primarily on genetic mutations or transcriptomic alterations, whereas the contribution of histone-modifying enzymes, including histone acetyltransferases, methyltransferases, kinases, and ubiquitin ligases, has received comparatively less attention. Furthermore, the mechanisms through which histone modification writers, erasers, and readers coordinate to regulate gene expression programs in uterine cells are not yet fully understood. Another major knowledge gap lies in determining how histone modification patterns differ between benign UFs and malignant LMS, and whether these epigenetic signatures contribute to tumor progression, cellular plasticity, or therapeutic resistance.

Additionally, although epigenetic therapies targeting histone-modifying enzymes have demonstrated promise in other malignancies [97,98,99], their potential application in USMTs remains largely unexplored [22]. Understanding how histone modification pathways contribute to tumor biology may provide valuable insights into novel diagnostic biomarkers and therapeutic strategies. In particular, identifying tumor-specific chromatin regulatory mechanisms may help distinguish benign from malignant smooth muscle tumors and reveal potential targets for precision medicine.

The purpose of this narrative review is therefore to provide current knowledge on histone modification pathways and their roles in uterine tumor biology. Specifically, this review aims to (1) summarize the major classes of histone modifications and their regulatory enzymes, including writers, erasers, and readers; (2) discuss emerging evidence linking histone modification dysregulation to the pathogenesis of UFs and uLMS; and (3) highlight existing knowledge gaps and future research directions. By integrating findings from molecular, epigenetic, and tumor biology studies, this review seeks to provide a comprehensive framework for understanding how histone modifications contribute to USMT development and may serve as potential targets for novel therapeutic interventions.

## 2. Histone Modifications in Uterine Fibroids

Beyond genetic susceptibility, increasing evidence highlights epigenetic dysregulation as a key mechanism in UF pathogenesis. Emerging evidence indicates that histone modifications play a central role in the epigenetic regulation of UFs, influencing gene expression programs that drive smooth muscle cell proliferation, ECM accumulation, and tumor growth. Histone-modifying enzymes, including acetyltransferases, deacetylases, methyltransferases, and bromodomain-containing readers, coordinate these modifications, linking chromatin dynamics with hormone signaling, developmental pathways, and cellular stress responses.

### 2.1. Histone Modifications: Writers in Uterine Fibroids

Histone methyltransferases, particularly EZH2, contribute to UF development by depositing repressive H3K27me3 marks on promoters of DNA repair genes (RAD51, BRCA1) and other target genes. Overexpression of EZH2 impairs DNA repair capacity and promotes cell proliferation in UF cells, while pharmacologic inhibition of EZH2 restores DNA repair gene expression, induces cell-cycle arrest, and suppresses UF growth [100]. Moreover, EZH2 activation enhances Wnt/β-catenin signaling by upregulating Wnt ligands (e.g., WNT5A, WNT9A) and nuclear β-catenin, driving proliferation and ECM accumulation. Treatment with methyl jasmonate (MJ) or EZH2 inhibitors reverses these effects, highlighting a key epigenetic mechanism that regulates UF cell behavior [101,102]. Collectively, EZH2-mediated H3K27me3 reprograms DNA repair and Wnt/β-catenin signaling network, thereby promoting proliferation and ECM remodeling in UFs.

MLL1 is a histone methyltransferase that catalyzes H3K4 methylation, a mark associated with transcriptional activation. In developmental exposure models (e.g., diethylstilbestrol, DES), MLL1 plays a central role in epigenetic reprogramming of myometrial stem cells (MMSCs). Through coordinated chromatin remodeling and DNA hypomethylation, MLL1 activates estrogen-responsive genes (ERGs) and inflammatory-responsive genes (IRGs) [103]. This persistent epigenetic reprogramming creates a hyper-estrogenic and pro-inflammatory cellular state, increasing susceptibility to UF development later in life. Moreover, these reprogrammed cells exhibit altered secretory profiles that can influence neighboring cells via paracrine signaling, amplifying disease progression.

The SRCAP chromatin remodeling complex regulates deposition of the histone variant H2A.Z, which is essential for proper chromatin organization and transcriptional regulation. In UFs, mutations in SRCAP components (e.g., *YEATS4*, *ZNHIT1*) disrupt H2A.Z incorporation, leading to altered chromatin accessibility and transcriptional dysregulation [104]. Defective H2A.Z deposition results in increased accessible chromatin state at transcription start sites, activation of embryonic stem cell–like gene programs, and epigenetic instability, ultimately contributing to aberrant cellular differentiation and tumorigenesis.

Genome-wide analyses have identified widespread alterations in histone modifications through profiling of active chromatin marks (H3K27ac, H3K4me3, and H3K4me1) in matched myometrium and UF tissues [105,106]. These studies demonstrated that the majority of epigenomic alterations in UFs occur at distal enhancer regions, suggesting a key role in reprogramming cell identity and transcriptional networks. Notably, HOXA13 was identified as a potential tumorigenic regulator influencing ECM-related gene expression in UFs [105]. A separate study showed that changes in H3K27ac disrupt enhancer activity, resulting in the activation of oncogenes and repression of tumor suppressor genes [107]. Similarly, altered H3K4me3 distribution affects promoter activity and transcriptional regulation, contributing to dysregulation of key signaling pathways, including Wnt/β-catenin and TGF-β in UFs [108]. Collectively, these epigenetic alterations reprogram the transcriptome, promoting aberrant cell proliferation, ECM remodeling, and the progression of UFs (Table 1).

### 2.2. Histone Deacetylases: Erasers in Uterine Fibroids

HDACs remove acetyl groups from histones, thereby regulating chromatin compaction and transcription. In UFs, HDAC1, HDAC3, and HDAC6 are upregulated, contributing to dysregulated gene expression and UF growth [109,111]. HDAC6, in particular, enhances estrogen receptor α (ESR1) stability and signaling, linking epigenetic regulation with hormone-driven proliferation [109]. More broadly, HDAC activity promotes transcription of genes involved in cell-cycle progression, ECM production, and TGF-β signaling pathways [111]. Pharmacologic inhibition of HDACs (e.g., SAHA) results in reduced cell proliferation, induction of cell-cycle arrest, decreased ECM components (fibronectin and collagen I), and suppression of TGF-β3 signaling, thereby limiting UF growth. Collectively, HDAC dysregulation links chromatin remodeling with estrogen signaling, ECM production, and proliferative pathways in UFs.

### 2.3. Histone Modifications: Readers in Uterine Fibroids

**Figure 2:**
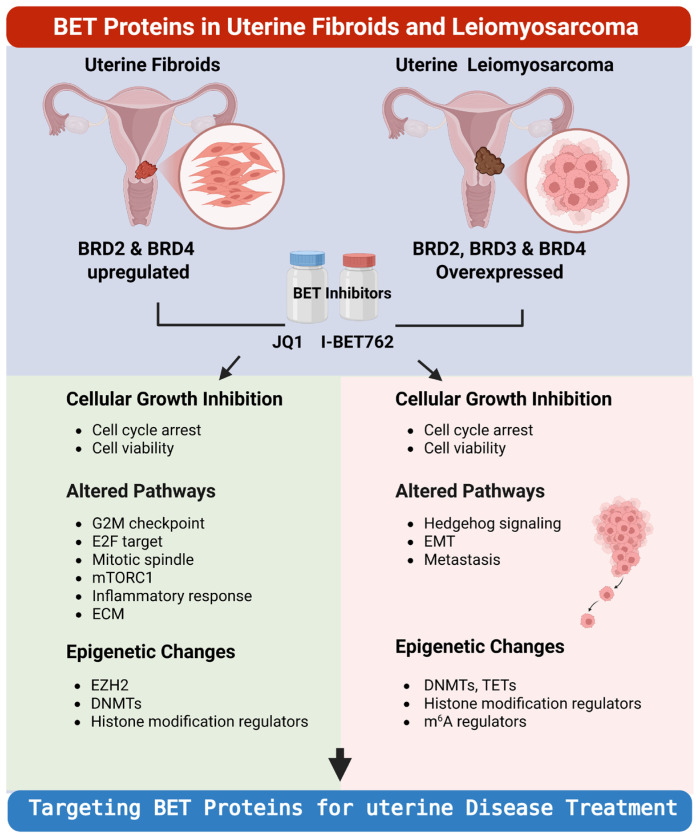
The Role and Mechanisms of BET proteins in Uterine Fibroids and Leiomyosarcoma. This figure illustrates the differential roles of BET family proteins in UFs and uLMS, as well as the therapeutic impact of BET inhibition. In UFs, BRD2 and BRD4 are upregulated and are associated with regulation of cell-cycle progression and reduced proliferation, along with alterations in pathways such as G2/M checkpoint control, E2F targets, mitotic spindle organization, mTORC1 signaling, inflammatory response, and ECM remodeling [115]. These changes are accompanied by epigenetic modifications involving EZH2, DNMTs, and other histone regulators. In uLMS, BRD2, BRD3, and BRD4 are overexpressed and contribute to tumor progression through activation of oncogenic pathways, including Hedgehog signaling, EMT, and metastasis. Treatment with BET inhibitors such as JQ1 and I-BET762 induces cell-cycle arrest and suppresses proliferation, in part by disrupting transcriptional regulation and epigenetic processes, including DNA and RNA methylation mediated by enzymes such as DNMTs, TETs, and m^6^A regulators [116]. Overall, the Figure highlights the central role of BET proteins in driving transcriptional and epigenetic programs in both benign and malignant USMTs and supports their potential as therapeutic targets.

BET family proteins, including BRD2 and BRD4, recognize acetylated histones and regulate transcriptional elongation. In UFs, these proteins are dysregulated and contribute to activation of multiple oncogenic pathways, including E2F, NF-κB, and mTORC1 signaling [115]. BET inhibition (e.g., JQ1, I-BET762) results in reduced cell viability, induction of cell-cycle arrest, and decreased ECM gene expression, reflecting suppression of UF growth and fibrotic remodeling (Figure 2).

**Figure 3:**
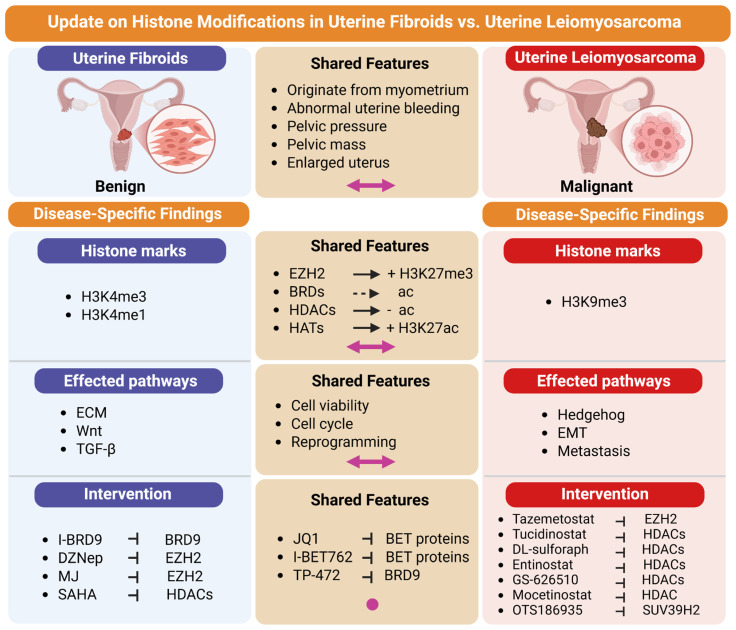
Update on Epigenetic and Signaling Pathway Landscape of Uterine Fibroids and Leiomyosarcoma. This figure summarizes the disease-specific findings and shared features in histone modifications and associated molecular pathways between UFs and uLMS. Both tumors originate from the myometrium and share common clinical features, including abnormal uterine bleeding, pelvic pressure, and pelvic mass, as well as overlapping molecular characteristics such as alterations in histone marks (e.g., H3K27ac and H3K27me3) and involvement of BET proteins, including BRD2, 4, 9. In addition, both conditions exhibit dysregulation of fundamental processes such as cell viability, cell-cycle control, and reprogramming, and may be targeted by epigenetic therapies including BET inhibitors (BETi), BRD9 inhibitors, HDAC inhibitors (HDACi), and EZH2 inhibitors. However, key disease-specific findings distinguish the benign nature of UFs from the malignant behavior of uLMS. In UFs, pathway alterations are primarily associated with ECM remodeling, Wnt signaling, and TGF-β pathway. In contrast, uLMS is characterized by activation of oncogenic pathways such as Hedgehog signaling, EMT, and metastasis. These distinctions highlight both shared epigenetic mechanisms and disease-specific pathways that may inform targeted therapeutic strategies.

BRD9 is an epigenetic reader that binds acetylated histones and regulates transcriptional programs. In UFs, BRD9 is upregulated and plays a critical role in maintaining tumorigenic transcriptional networks. Pharmacologic inhibition of BRD9 (e.g., I-BRD9, TP-472) leads to epigenomic and epitranscriptomic reprogramming, affecting pathways related to cell-cycle progression, inflammatory response, and ECM regulation [113,114]. Functionally, BRD9 inhibition induces apoptosis, cell-cycle arrest, and reduced ECM deposition, suppressing UF growth (Figure 3). Collectively, BRD9 sustains tumorigenic transcriptional and epigenetic programs that support proliferation, inflammatory signaling, and ECM remodeling in UFs.

### 2.4. Integrated Crosstalk Between Histone Modifications, Epigenetic Regulation, Signaling Pathways, and Epitranscriptomic Mechanisms in UFs

Histone modifications in UFs function within a highly interconnected regulatory network rather than as isolated events. They dynamically interact with other epigenetic mechanisms, including DNA methylation, chromatin remodeling, enhancer–promoter looping, and non-coding RNA pathways, to coordinate transcriptional programs that drive UF initiation and progression. For example, H3K27me3 mediated by EZH2 represses key DNA repair genes such as RAD51 and BRCA1, thereby altering genomic stability while creating a permissive chromatin environment for additional epigenetic abnormalities, including aberrant DNA methylation patterns [100]. Likewise, dysregulated activating marks such as H3K27ac, H3K4me1, and H3K4me3 remodel enhancer landscapes and promoter accessibility, disrupting developmental gene networks including HOX family genes that are critical for uterine tissue homeostasis [105,107].

These chromatin alterations are closely integrated with tumor subtype, major signaling pathways implicated in UF biology [105]. Epigenomic remodeling enhances transcriptional responses to profibrotic and proliferative signals such as TGF-β and Wnt/β-catenin, thereby promoting ECM accumulation, tumor growth, and long-term maintenance [105,107,108]. Activated β-catenin signaling further cooperates with HDACs and estrogen receptor pathways to induce genes associated with proliferation and survival, including cyclin D1 and c-Myc [110]. Thus, histone modifications serve as central mediators that translate hormonal, developmental, and environmental signals into persistent pathological gene-expression programs.

RNA modifications, collectively known as the epitranscriptome, play increasingly recognized roles in female cancer and reproductive diseases [117,118,119]. Beyond transcriptional regulation, histone modifications also intersect with epitranscriptomic control at the RNA level. Emerging evidence underscores extensive crosstalk between histone modifications and RNA epitranscriptomic regulation, forming an integrated, multilayered network that coordinates gene expression in tumors [120]. Histone-modifying enzymes can influence RNA modification pathways by regulating the transcription of epitranscriptomic “writers,” “readers,” and “erasers.” Conversely, RNA modifications, such as 5-methylcytidine (m^5^C) and N^6^-methyladenosine (m^6^A), can modulate chromatin states by altering RNA stability, translation efficiency, and RNA–protein interactions [121,122,123]. In UFs, bromodomain-containing proteins such as BRD9 recognize acetylated histones while simultaneously influencing transcription of RNA-modifying enzymes, including components of the m^6^A machinery, thereby linking chromatin state to RNA methylation, RNA stability, and translational efficiency [113,114]. This coordinated regulation of transcriptional and post-transcriptional processes affects core UF phenotypes such as cell proliferation, apoptosis resistance, differentiation, and ECM remodeling and these processes establish a dynamic and reciprocal regulatory axis that fine-tunes gene expression in a context-dependent manner.

Environmental exposures are increasingly recognized as important contributors to UF pathogenesis, acting through endocrine disruption, epigenetic reprogramming, and inflammatory signaling [124,125,126]. Developmental exposure to endocrine-disrupting chemicals can reprogram histone marks through enzymes such as MLL1, establishing persistent epigenetic memory that alters downstream RNA processing and cellular behavior later in life [101,103,127]. Such findings support the concept that UFs arise through multilayered interactions between inherited susceptibility and environmentally induced epigenetic reprogramming.

Collectively, epigenetic writers (e.g., EZH2, MLL1, SRCAP), erasers (HDACs), and readers (BRDs) form a coordinated regulatory circuitry that governs chromatin architecture and gene expression in UFs. Dysregulation of this network contributes to enhanced proliferation and survival, excessive ECM deposition, aberrant hormonal and inflammatory signaling, impaired differentiation, and genomic/epigenetic instability (Table 1). These insights underscore the central role of integrated epigenetic regulation in UF pathogenesis and highlight epigenetic modifiers as promising targets for non-hormonal therapeutic strategies.

## 3. Histone Modifications in Uterine Leiomyosarcoma

ULMS is a rare but highly aggressive malignancy of smooth muscle origin, characterized by rapid proliferation, genomic instability, and poor clinical outcomes. While genetic alterations contribute to tumor initiation, growing evidence suggests that epigenetic dysregulation, particularly histone modifications, plays a central role in uLMS pathogenesis. Aberrant activity of histone-modifying enzymes, including deacetylases, acetyltransferases, methyltransferases, and bromodomain-containing readers, reprograms chromatin states, regulates transcriptional networks, and contributes to the malignant phenotype.

### 3.1. Histone Modification Writers in uLMS

Histone-modifying “writer” enzymes play a central role in shaping the epigenetic landscape of uLMS by dynamically regulating chromatin accessibility and transcriptional activity. Among HATs, HAT1 has emerged as a key oncogenic regulator that promotes transcriptional activation through histone acetylation and open chromatin formation. Functionally, HAT1 operates downstream of oncogenic signaling pathways, and its suppression, such as through combined pazopanib and hyperthermia treatment, leading to reduced histone acetylation, inhibition of tumor growth, and attenuation of proliferative signaling [128]. Clinically, elevated HAT1 expression is associated with aggressive tumor behavior and poorer outcomes, supporting its potential as both a prognostic biomarker and a therapeutic target in uLMS.

In parallel, histone methyltransferases (HMTs) contribute to transcriptional regulation through context-dependent chromatin compaction and gene silencing or activation. SUV39H2, a key H3K9 methyltransferase, is overexpressed in uLMS and facilitates DNA damage repair by promoting γH2AX recruitment; its inhibition disrupts double-strand break repair and enhances sensitivity to PARP inhibitors, highlighting a synthetic lethality strategy [129]. Metabolic reprogramming further intersects with histone methylation, as fatty acid synthase (FASN)-driven lipogenesis alters histone modification landscapes, including increased H3K9me3 and H3K27ac, thereby linking metabolic pathways to epigenetic control of gene expression [130]. Additionally, EZH2, the catalytic component of the PRC2 complex responsible for H3K27 trimethylation, mediates transcriptional repression of tumor suppressor genes; its inhibition induces apoptosis and exhibits synergistic antitumor effects when combined with histone deacetylase inhibitors [131]. Collectively, these findings underscore the coordinated roles of HATs and HMTs in driving epigenetic reprogramming and tumor progression in uLMS (Table 2).

### 3.2. Histone Modification Erasers in uLMS

HDACs are key “eraser” enzymes that regulate chromatin structure by removing acetyl groups from histone lysine residues, leading to chromatin condensation and transcriptional repression. In uLMS, Class I HDACs (HDAC1, HDAC2, and HDAC3) are consistently upregulated and contribute to oncogenic transcriptional programs that support tumor proliferation and survival. Pharmacologic inhibition of these enzymes, including agents such as tucidinostat and entinostat, suppresses cell proliferation and induces broad transcriptomic reprogramming, highlighting their therapeutic relevance [131,135]. In addition, HDAC5, HDAC7, and HDAC9 have been associated with prognostic significance in uLMS, where lower expression correlates with improved disease-free survival, underscoring their potential value as clinical biomarkers [133]. Notably, HDAC8 serves as a lineage-specific marker of smooth muscle differentiation, aiding in the pathological distinction between uLMS and endometrial stromal tumors [132].

Functionally, HDAC inhibition exerts broad epigenomic and immunoregulatory effects beyond transcriptional derepression. HDAC inhibitors increase chromatin accessibility and enhance H3K27 acetylation at regulatory elements, thereby activating enhancer landscapes and altering gene expression programs. They also induce transcriptional activation of transposable elements and endogenous retroviruses (ERVs), contributing to double-stranded RNA formation and modulation of innate immune signaling pathways. However, despite increased ERV expression, immune activation may be attenuated by RNA editing mechanisms such as ADAR-mediated modification, reflecting complex epigenetic–epitranscriptomic interplay in uLMS [136]. Collectively, these findings highlight HDACs as central regulators of chromatin dynamics, tumor progression, and immune modulation in uLMS, and support HDAC-targeted strategies as promising therapeutic approaches.

### 3.3. Histone Modification Readers in uLMS

Histone reader proteins recognize acetylated lysine residues on histones and translate epigenetic marks into transcriptional programs that regulate gene expression. In uLMS, BRD9 is overexpressed and functions as a critical regulator of oncogenic transcriptional networks, including MYC, KRAS, NF-κB, and mTOR signaling pathways. Functional studies have shown that inhibition of BRD9 induces apoptosis and cell-cycle arrest, highlighting its importance in tumor maintenance and progression [134].

Similarly, members of the BET family, including BRD2, BRD3, and BRD4, are frequently upregulated and contribute to transcriptional programs associated with epithelial–mesenchymal transition (EMT), Hedgehog signaling, and broader tumor progression pathways. Pharmacologic BET inhibition using compounds such as JQ1 and I-BET762 has been shown to suppress tumor cell proliferation and reprogram epigenetic regulatory networks [116]. Collectively, these findings underscore histone reader proteins as key epigenetic mediators that connect chromatin state to oncogenic signaling and represent promising therapeutic vulnerabilities in aggressive cancers such as uLMS (Figure 2, Table 2).

ATRX is increasingly recognized as an important epigenetic regulator in uterine tumors. Recurrent ATRX mutations and loss of expression have been reported [138,139] in uLMS and UFs, respectively, while mechanistic studies in other systems show that ATRX cooperates with DAXX to deposit histone variant H3.3 at telomeric and heterochromatic regions [139,140] and recognizes repressive histone marks such as H3K9me3 [141]. Loss of ATRX can disrupt heterochromatin integrity, alter histone modification patterns, impair telomere maintenance, and promote transcriptional dysregulation, thereby contributing to malignant progression in USMTs [142,143] (Table 2).

### 3.4. Crosstalk Between Histone Modifications and Other Epitranscriptomic Regulation in uLMS

The epigenetic landscape of uLMS is highly interconnected, with histone modifications serving as a central regulatory hub that interfaces with multiple layers of epigenetic control. Rather than functioning in isolation, histone-based chromatin regulation is tightly coordinated with DNA methylation patterns, non-coding RNAs, and epitranscriptomic networks, collectively shaping transcriptional programs that govern tumor initiation, progression, and therapeutic resistance. This multilayered epigenetic crosstalk establishes a dynamic regulatory framework in which alterations in one pathway can propagate across others, thereby reinforcing malignant phenotypes.

#### 3.4.1. Crosstalk Between Histone Modifications and DNA Methylation and microRNA Regulation in uLMS

Crosstalk between histone modifications and other epigenetic regulatory layers plays a central role in the pathobiology of uLMS, where coordinated disruptions in chromatin structure, DNA methylation, and non-coding RNA networks converge to drive malignant gene expression programs. Histone post-translational modifications (PTMs), particularly acetylation and methylation, regulate chromatin accessibility through the action of “writer,” “eraser,” and “reader” proteins, thereby influencing transcriptional activation or repression of genes involved in cell-cycle control, DNA repair, and apoptosis. In uLMS, RNA-seq revealed the downregulation of DNMT3A, DNMT3B, DNMT1, TET1, and TET2 in uLMS cells in response to targeted inhibition of BET proteins [116] dysregulation of histone-modifying enzymes such as EZH2-mediated H3K27 trimethylation and HDAC-dependent deacetylation contributes to a repressive chromatin landscape that cooperates with DNA methylation changes to silence tumor suppressor genes and reinforce oncogenic signaling pathways. Integrated epigenomic analyses further demonstrate that uLMS exhibits distinct DNA methylation signatures compared with benign myometrial tissue and other sarcoma subtypes, underscoring the tight functional coupling between histone-based chromatin states and DNA methylation in shaping tumor identity and aggressiveness [144].

Beyond DNA methylation, non-coding RNAs, especially microRNAs, add an additional regulatory layer that interacts bidirectionally with histone modifications in uLMS. miRNAs can modulate the expression of chromatin-modifying enzymes (such as DNMTs, HDACs, and histone methyltransferases), while histone marks at miRNA gene promoters determine their transcriptional output, creating reinforcing feedback loops that stabilize malignant phenotypes [145,146]. In uLMS, targeted inhibition of HDACs induced differentially expressed genes that are putative targets of multiple miRNAs [135]. Accumulated data suggest that this multi-layered epigenetic crosstalk contributes to tumor heterogeneity, therapeutic resistance, and disease progression by enabling dynamic reprogramming of chromatin states in response to oncogenic and microenvironmental stress [132,147]. Collectively, these findings highlight the epigenome in uLMS as an interconnected regulatory network rather than isolated pathways, supporting the rationale for combinatorial epigenetic therapies targeting both histone modifiers and DNA methylation machinery [148].

#### 3.4.2. Crosstalk Between Histone Modifications and RNA Epitranscriptomic Regulation in uLMS

In uLMS, accumulating evidence further supports a bidirectional interplay between histone modifications and RNA epitranscriptomic regulation. Pharmacologic inhibition of the m^6^A demethylase FTO using Dac51 significantly alters the expression of histone acetylation–associated genes, including HDAC1, HDAC10, SIRT1, and SIRT2, demonstrating that RNA methylation machinery can directly modulate histone modification pathways [149]. Conversely, inhibition of HDACs increases chromatin accessibility and enhances H3K27ac enrichment at regulatory regions, leading not only to transcriptional activation but also to increased expression of transposable elements and double-stranded RNAs that are subject to RNA editing and epitranscriptomic regulation [136].

In addition, BET inhibition (e.g., JQ1 and I-BET762) alters the expression of key m^6^A regulators such as FTO, YTHDC2, and IGF2BP1, supporting a direct role for histone “readers” in reprogramming the epitranscriptome in uLMS [116,134]. In addition, metabolic reprogramming contributes to this crosstalk: fatty acid synthase (FASN)-driven lipid metabolism reshapes histone methylation landscapes, such as increased H3K9me3, while simultaneously influencing RNA regulatory networks [130] (Figure 2 and Figure 3, Table 2).

Taken together, these findings support a model in which histone modifications and other regulators and pathways in a coordinated and context-dependent manner to regulate gene expression, tumor progression, and therapeutic response in malignant uterine tumors.

## 4. Clinical Implications and Therapeutic Potential

Advances in understanding the epigenetic landscape of USMTs, including benign UFs and malignant uLMS, have revealed important clinical implications. Histone modifications influence gene expression programs that regulate cell proliferation, apoptosis, ECM production, and DNA repair. Consequently, targeting epigenetic regulators has emerged as a promising therapeutic strategy. In addition, histone modification patterns and the expression of histone-modifying enzymes may serve as valuable biomarkers for diagnosis, prognosis, and therapeutic decision-making.

### 4.1. Histone Modification Inhibitors

Pharmacological targeting of histone-modifying enzymes represents a major area of interest in epigenetic therapy for UFs and uLMS/LMS. Among these agents, histone deacetylase (HDAC) inhibitors have received considerable attention due to their ability to restore balanced histone acetylation and reactivate silenced tumor suppressor genes. In UFs, the HDAC inhibitor SAHA has been shown to upregulate tumor suppressor gene expression, highlighting its role in reversing epigenetic repression [107]. HDAC inhibitors such as Tucidinostat and DL-sulforaphane, as well as Entinostat increase histone acetylation, relax chromatin structure, and suppress tumor growth by inducing apoptosis, cell- cycle arrest in uLMS and uterine sarcoma (US), respectively [131,135], and inhibiting processes such as wound healing in US [131]. These findings underscore the capacity of HDAC inhibition to disrupt oncogenic transcriptional programs and alter tumor-supportive signaling pathways. In a Phase II study of metastatic LMS (Including a uLMS subset) resistant to prior gemcitabine therapy, mocetinostat (an HDACi) plus gemcitabine showed modest activity with mainly stable disease and manageable toxicities, although a rare significant pericardial adverse event occurred [137].

Another important class of epigenetic therapeutics includes histone methyltransferase (HMT) inhibitors, particularly those targeting Enhancer of Zeste Homolog 2 (EZH2), a key catalytic component of the polycomb repressive complex 2 (PRC2). In UFs, pharmacologic inhibition of EZH2 using agents such as DZNep or methyl jasmonate disrupts EZH2 activity, leading to epigenetic reprogramming and suppression of cell proliferation [102,112]. Mechanistically, EZH2 inhibition reduces H3K27me3-mediated transcriptional repression, thereby restoring expression of genes involved in tumor suppression and cellular homeostasis. In US, the EZH2 inhibitor Tazemetostat similarly inhibits H3K27 trimethylation, resulting in apoptosis and cell-cycle arrest [131], further supporting the therapeutic relevance of targeting histone methylation in malignant uterine tumors.

In addition, emerging evidence highlights a critical role for the histone methyltransferase SUV39H2 in uLMS. SUV39H2 is significantly overexpressed in uLMS compared with normal myometrium and UFs and contributes to double-strand DNA break repair through recruitment of γH2AX. Pharmacologic inhibition using OTS186935 suppresses uLMS cell viability and impairs DNA damage repair, as evidenced by reduced γH2AX accumulation and chromatin-level alterations. Notably, SUV39H2 inhibition induces synthetic lethality when combined with the PARP inhibitor olaparib, resulting in enhanced tumor cell death in vitro and significantly improved antitumor efficacy in vivo. These findings identify SUV39H2 as a key epigenetic regulator linking chromatin modification to DNA repair dependency and highlight its potential for combination strategies targeting DNA damage response pathways in uLMS [129].

In addition to writers and erasers of histone modifications, epigenetic “readers” such as bromodomain and extra-terminal domain (BET) proteins and BRD9 have emerged as critical regulators of transcriptional programs. In UFs, inhibition of BRD9 using compounds such as TP-472 and I-BRD9 disrupts the recognition of acetylated histones and BRD9-dependent transcription, leading to reduced proliferation, induction of apoptosis, decreased ECM deposition, and broad reprogramming of both the epigenome and epitranscriptome [113,114]. Similarly, BET inhibitors such as JQ1 and I-BET762 block the interaction between BET proteins and acetylated histones, thereby suppressing oncogenic transcriptional programs. In UFs, BET inhibition impacts pathways associated with proliferation, survival, inflammatory signaling, and ECM accumulation [115] (Figure 3). In contrast, in uLMS, BET inhibition suppresses transcriptional addiction pathways and downregulates RNA modification regulators, including FTO, YTHDC2, and IGF2BP1 [116], highlighting a novel link between chromatin regulation and the epitranscriptome (Figure 3, Table 3).

Collectively, these studies demonstrate that targeting histone modification machinery, including HDACs, EZH2, SUV39H2, BET proteins, and BRD9, can effectively modulate gene expression, inhibit tumor growth, and induce apoptosis in both benign and malignant uterine smooth muscle tumors. Moreover, the observed synergy between different classes of epigenetic inhibitors suggests that combinatorial therapeutic strategies may offer enhanced efficacy, particularly in aggressive diseases such as uLMS (Figure 3).

### 4.2. Epigenetic Biomarkers for Diagnosis, Prognosis, and Treatment Stratification

Epigenetic alterations represent a promising class of biomarkers for the diagnosis and clinical management of uterine smooth muscle tumors. Abnormal expression of histone-modifying enzymes, including HDACs, histone acetyltransferases, and bromodomain-containing proteins, has been consistently observed in benign UFs and malignant LMS [107,115]. These epigenetic differences reflect distinct transcriptional and chromatin states and may provide valuable tools for distinguishing histologically ambiguous uterine mesenchymal tumors, thereby improving diagnostic accuracy.

In addition to diagnostic applications, specific epigenetic regulators have emerged as potential prognostic markers. For example, dysregulated expression of HDAC isoforms has been linked to tumor aggressiveness and clinical outcomes in LMS, where HDAC inhibition induces apoptosis and cell-cycle arrest, suggesting a functional association with disease progression [131]. Similarly, overexpression of bromodomain proteins such as BRD4 and BRD9 has been associated with activation of oncogenic transcriptional programs. In UFs, BRD9-driven transcription contributes to proliferation and ECM accumulation, while its inhibition suppresses tumor growth and reprograms the epigenome [113,114]. In malignant settings, BET protein activity has been linked to transcriptional addiction pathways and regulation of RNA modification machinery, further supporting their role in aggressive tumor behavior and poor clinical outcomes [116]. Together, these findings highlight the potential of epigenetic regulators as biomarkers for risk stratification and as predictors of response to targeted therapies.

**Figure 4:**
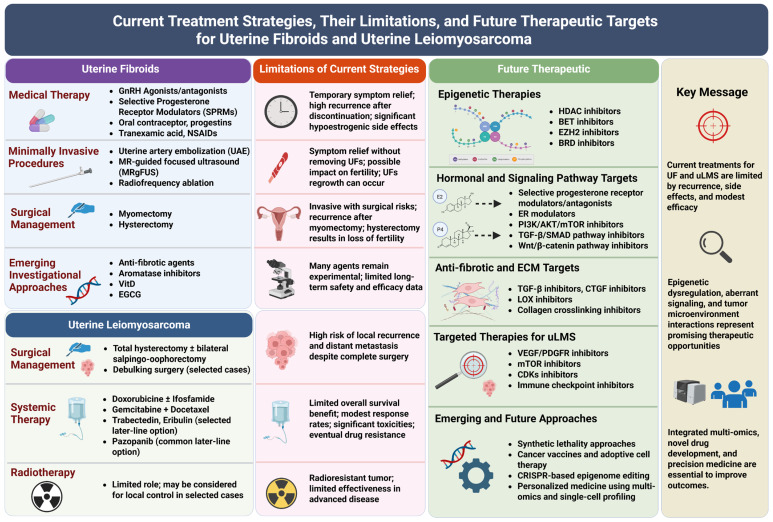
Current treatment strategies, limitations, and emerging therapeutic targets for uterine fibroids (UF) and uterine leiomyosarcoma (uLMS). Current management of UF includes medical therapies, minimally invasive procedures, and surgical interventions, whereas uLMS treatment primarily relies on surgery, chemotherapy, targeted therapy, and selected radiotherapy. Despite these approaches, major challenges remain, including recurrence, infertility, treatment resistance, systemic toxicity, and limited efficacy in advanced disease. The figure highlights emerging therapeutic opportunities targeting epigenetic regulators, hormone and signaling pathways, ECM remodeling, and tumor microenvironment interactions, as well as novel approaches such as RNA-based therapeutics, immune checkpoint inhibition, CRISPR-based epigenome editing, and precision medicine strategies guided by multi-omics and single-cell analyses.

Furthermore, integrative epigenetic signatures encompassing histone modification patterns, DNA methylation landscapes, non-coding RNA expression profiles, and RNA epitranscriptomics provide a more comprehensive understanding of tumor heterogeneity. Such multi-layered epigenetic profiling can capture dynamic regulatory networks that underlie tumor initiation and progression. Incorporation of these biomarkers into molecular diagnostic frameworks may facilitate personalized treatment strategies, enable more precise classification of USMTs, and improve patient outcomes (Figure 4).

### 4.3. Challenges and Limitations of Translating Epigenetic Therapies

Despite promising preclinical results, several challenges remain in translating epigenetic therapies into clinical practice for uterine tumors. One major limitation is the lack of tumor specificity of many epigenetic drugs. Histone-modifying enzymes regulate gene expression across the entire genome, and their inhibition can affect both tumor cells and normal tissues, potentially leading to off-target effects and toxicity.

Another challenge involves the complexity and plasticity of epigenetic regulation. Histone modifications often interact with other regulatory mechanisms, including DNA methylation and non-coding RNAs, forming highly interconnected networks. As a result, inhibition of a single epigenetic enzyme may not be sufficient to produce durable therapeutic responses, as tumor cells may compensate through alternative pathways.

Additionally, tumor heterogeneity within uLMS may limit the effectiveness of epigenetic therapies, as different tumors may exhibit distinct epigenetic alterations and regulatory dependencies. Identifying reliable biomarkers to predict treatment response remains an important goal for future research.

Finally, while several epigenetic inhibitors have been approved for the treatment of other malignancies, clinical trials specifically evaluating these agents in USMTs remain limited. Further studies are required to determine optimal drug combinations, dosing strategies, and patient selection criteria.

Overall, continued investigation of histone modification pathways and their interactions with other epigenetic mechanisms will be critical for translating epigenetic discoveries into effective clinical interventions for USMTs (Figure 4).

## 5. Future Directions

Despite significant advances in understanding the role of epigenetic regulation in USMTs, including benign UFs and malignant uLMS, many aspects of histone modification–mediated regulation remain incompletely understood. Future research efforts should focus on integrating emerging technologies and systems-level approaches to better characterize the epigenetic landscape of these tumors and to translate this knowledge into improved diagnostic and therapeutic strategies.

### 5.1. Epigenomic Profiling in Uterine Leiomyosarcoma

One of the key gaps in the field is the imbalance in epigenomic knowledge between benign and malignant disease. Despite significant advances in defining genome-wide histone modification landscapes in UFs, comparable epigenomic profiling in uLMS remains largely incomplete. Given the marked biological and clinical differences between these entities, systematic mapping of active and repressive histone marks in uLMS is urgently needed to define malignant-specific chromatin states. Integrative enhancer–promoter analyses, particularly at distal regulatory elements, would be critical for identifying uLMS-specific transcriptional circuits that drive oncogenic progression and metastatic potential. In addition, comparative epigenomic studies between UF and uLMS could uncover key points in chromatin reprogramming associated with malignant phenotype, including shifts in enhancer activation, promoter remodeling, and Polycomb-mediated repression. Coupling these datasets with chromatin accessibility (ATAC-seq), transcription factor occupancy, and 3D genome architecture would further enable reconstruction of regulatory networks underlying tumor aggressiveness. Ultimately, such comprehensive epigenomic profiling may reveal disease-specific vulnerabilities and support the development of targeted epigenetic therapies for uLMS.

### 5.2. Multi-Omics Integration to Understand Histone Regulation in USMTs

One of the key priorities for future research is the integration of multi-omics approaches to comprehensively characterize histone regulation in uterine smooth muscle tumors. Histone modifications interact with numerous molecular layers, including DNA Methylation, Transcriptomics, Proteomics, and Metabolomics, forming complex regulatory networks that shape tumor behavior. Integrating these datasets can provide a more complete understanding of how epigenetic mechanisms coordinate transcriptional programs involved in tumor initiation, growth, and progression, particularly in uLMS, where such analyses remain largely lacking.

For example, combining histone modification profiling with genome-wide transcriptomic data can identify regulatory elements that control key oncogenic pathways. Similarly, integration with DNA methylation and chromatin accessibility data may reveal how different epigenetic layers cooperate to regulate gene expression. Such comprehensive analyses could help identify critical epigenetic drivers of tumor development and uncover new biomarkers for early detection and prognosis. Multi-omics strategies may also identify epigenetic markers that distinguish benign UFs from malignant uLMS, as well as reveal mechanisms underlying therapy resistance in uLMS.

In addition, the relationship between histone modifications and alternative splicing regulation in USMTs remains poorly understood, particularly in the context of GWAS-identified risk loci. Future integrative epigenomic, transcriptomic, and GWAS-based analyses are needed to clarify how chromatin states and histone modification landscapes influence splice-site selection and gene regulatory networks in USMT pathogenesis.

### 5.3. Advanced Technologies: Single-Cell Epigenomics and CRISPR-Based Epigenetic Editing

Recent technological advances are providing powerful tools to dissect epigenetic regulation at unprecedented resolution [151]. Single-cell epigenomics allows researchers to analyze chromatin accessibility, histone modifications, and gene expression at the level of individual cells [152,153]. This approach is particularly valuable for studying tumor heterogeneity within uterine smooth muscle tumors, where distinct cell populations, including tumor cells, stem-like cells, fibroblasts, and immune cells, may contribute differently to disease progression [2,154,155,156,157].

Single-cell epigenomic analyses can reveal cell-type–specific histone modification patterns and identify regulatory circuits that drive tumor growth or therapeutic resistance. These studies may also uncover rare subpopulations of tumor cells with enhanced proliferative or metastatic potential, providing important insights into tumor evolution and disease progression [158,159,160].

Another promising technology is CRISPR-based epigenetic editing, which enables targeted manipulation of epigenetic marks without altering the underlying DNA sequence [161,162,163]. By using modified CRISPR systems fused to epigenetic modifiers, researchers can selectively add or remove histone marks at specific genomic loci [164,165]. This approach allows direct investigation of the functional roles of histone modifications in regulating gene expression and cellular phenotypes [164,165]. In the context of uterine smooth muscle tumors, CRISPR-based epigenetic editing could be used to determine how specific histone marks influence oncogenic pathways, ECM production, or hormone responsiveness.

### 5.4. Personalized Therapy Approaches Targeting Epigenetic Modifications

As our understanding of epigenetic regulation in USMTs continues to expand, there is increasing interest in developing personalized therapeutic strategies that target tumor-specific epigenetic alterations [144]. Because epigenetic modifications are reversible, they represent attractive targets for therapeutic intervention. Identification of patient-specific epigenetic signatures, such as altered expression of histone-modifying enzymes or characteristic histone modification patterns, may allow clinicians to tailor treatments based on the molecular profile of individual tumors.

Personalized epigenetic therapy may involve the use of selective inhibitors targeting histone-modifying enzymes, such as HDACs or histone methyltransferases, either as monotherapies or in combination with conventional treatments. Additionally, combining epigenetic therapies with targeted therapies, immunotherapy, or hormone-based treatments may enhance therapeutic efficacy by simultaneously modulating multiple signaling pathways.

Future clinical studies will be essential to evaluate the safety, efficacy, and optimal combinations of epigenetic drugs in patients with uterine smooth muscle tumors. Ultimately, integrating epigenomic profiling into clinical decision-making may pave the way for precision medicine approaches that improve outcomes for patients with both benign and malignant USMTs (Figure 4).

## 6. Conclusions

USMTs represent a spectrum of diseases ranging from benign UFs to highly aggressive uLMS. Although these tumors arise from a common cellular origin, they display markedly different biological behaviors and clinical outcomes. Increasing evidence highlights the critical role of Histone Modification in shaping the epigenetic landscape that governs gene expression, cellular proliferation, differentiation, and tumor progression in uterine smooth muscle tissues.

### 6.1. Shared and Divergent Histone Modification Landscapes in Uterine Fibroids and Leiomyosarcoma

This review highlights both shared epigenetic architecture and disease-specific rewiring of histone modifications in UFs and uLMS (Figure 2 and Figure 3). In both tumor types, histone modification machinery converges on core regulators of chromatin accessibility and transcriptional control. Notably, HDACs, the methyltransferase EZH2 (catalyzing H3K27me3), and bromodomain (BRD) proteins that recognize acetylated lysines are commonly engaged, underscoring a shared dependency on dynamic acetylation–methylation balance. This epigenetic convergence is functionally reflected in overlapping biological outcomes, including regulation of cell-cycle progression and cell viability. Consistently, pharmacologic targeting of these shared regulators, such as BET inhibitors (e.g., JQ1, I-BET762) and HDAC inhibitors, induces cell-cycle arrest and transcriptional reprogramming in both UF and uLMS, indicating that chromatin reader–writer–eraser networks constitute a common therapeutic vulnerability.

Despite these similarities, the two diseases diverge markedly in the specific histone marks and regulatory pathways that dominate their epigenetic landscapes. In UF, histone modification patterns are more consistent with a benign, proliferative phenotype, characterized by histone mark enrichment and coordinated activation of pathways such as ECM remodeling, inflammatory signaling, Wnt signaling, and TGF-β pathway. Moreover, histone modification profiling shows distinct enriched region in UFs, and differential genes correlated with histone modification status. In contrast, uLMS exhibits a distinct chromatin state, highlighted by histone modification regulators including SUV39H2-driven H3K9me3 deposition, a hallmark of heterochromatin formation and transcriptional silencing. This shift is accompanied by activation of oncogenic and metastatic programs, including Hedgehog signaling, EMT, and metastasis-related pathways. Furthermore, while BET proteins are upregulated in both conditions, uLMS shows broader overexpression (BRD2/3/4) and deeper integration with additional epigenetic layers, including DNA methylation dynamics (DNMTs/TETs), and m^6^A RNA modification regulators. Together, these findings suggest that while UF and uLMS share core chromatin regulatory mechanisms, their downstream pathways and target gene networks diverge substantially. This divergence likely reflects differential amplification and integration of these epigenetic pathways into a coordinated, high plasticity epigenomic state that drives tumor aggressiveness.

These observations are primarily derived from current studies and available datasets. Further comprehensive and systematic investigations, including expanded cohort studies, multi-omics integration, and functional validation in USMTs, are required to confirm, refine, and extend these findings. Such studies will be essential to achieve a comprehensive understanding of the shared and divergent epigenetic mechanisms underlying UFs and uLMS, and to strengthen their translational relevance.

### 6.2. Epigenetic Regulation and Therapeutic Implications of Histone Modifications in Uterine Smooth Muscle Tumors

This narrative review summarizes current knowledge on the major classes of histone modifications, and the enzymes responsible for their regulation, such as writers, erasers, and readers. These epigenetic regulators collectively modulate chromatin structure and transcriptional activity, thereby influencing key signaling pathways involved in tumor development. In UFs, histone modifications often regulate hormone-responsive pathways, ECM production, and cellular proliferation, contributing to benign tumor growth. In contrast, uLMS exhibits extensive epigenetic reprogramming characterized by dysregulation of histone-modifying enzymes, aberrant chromatin remodeling, and activation of oncogenic signaling pathways that promote tumor aggressiveness, genomic instability, and metastatic potential.

Importantly, histone modifications function within a broader epigenetic network that includes DNA methylation, microRNA, long non-coding RNA–mediated regulation, and RNA modifications. The interplay among these mechanisms coordinates transcriptional programs that influence tumor initiation, growth, and progression. Understanding this complex regulatory network is essential for identifying molecular differences between benign and malignant USMTs and for uncovering novel therapeutic opportunities. Recent advances highlight the clinical relevance of targeting histone-modifying enzymes. Epigenetic inhibitors, including histone deacetylase inhibitors, histone methyltransferase inhibitors, and histone modification readers, have shown promising antitumor effects by restoring balanced chromatin states and disrupting oncogenic transcriptional programs.

Despite these advances, many aspects of histone-mediated regulation in USMTs remain poorly understood. Future research integrating multi-omics approaches, advanced epigenomic technologies, AI-driven computational analyses, and functional studies will be crucial to fully elucidating the role of histone modifications in tumor biology. Such efforts may ultimately enable the development of personalized epigenetic therapies, guided by AI–assisted biomarker discovery and patient stratification, to improve clinical outcomes for patients with both benign and malignant uterine smooth muscle tumors.

## Figures and Tables

**Table 1:** The Role of Histone Modifications in Uterine Fibroids.

Disease	Sample Type	Approach	Mechanism	Biological Effect	Publication Date	Ref.
Uterine fibroids	Human UF and matched normal myometrium tissues, and ELT-3 cells	Expression analysis of HDAC6 and estrogen receptor α in tissues; correlation analysis between gene expression levels; gene silencing of HDAC6 in ELT-3 cells to evaluate effects on estrogen signaling and cell proliferation	Upregulation of HDAC6 enhances stability and signaling of ESR1 (estrogen receptor α), linking histone deacetylase activity with estrogen signaling pathways that regulate UF growth.	HDAC6 expression correlates strongly with ERα levels in UF tissues, and silencing HDAC6 reduces ERα expression, weakens estrogen responsiveness, and suppresses UF cell proliferation	August 2011	[109]
Uterine fibroids	Human UF and matched adjacent MM tissues, and UF-derived cell cultures	Analysis of RAD51, BRCA1, and EZH2 protein levels using IHC and Western blot; pharmacologic inhibition of EZH2 in UF cells to examine effects on gene expression, histone modification, and cell proliferation	f EZH2 OE promotes H3K27me3 at promoters of DNA repair genes (RAD51 and BRCA1), suppressing their transcription and impairing DNA repair capacity in UF cells.	Reduced DNA repair activity contributes to UF development, while EZH2 inhibition restores RAD51 and BRCA1 expression, suppresses UF cell proliferation, and induces cell-cycle arrest.	March 2016	[100]
Uterine fibroids	Human UF tissues and matched MM, and primary UF/MM cell populations	Protein and RNA analyses; chemical inhibition/activation and genetic knockdown (KD) of β-catenin; treatment with estradiol and HDAC inhibitors to evaluate effects on UF cell phenotype	Enhanced nuclear β-catenin signaling in UFs activates downstream genes. This pathway shows crosstalk with estrogen signaling and HDACs, where estradiol promotes β-catenin nuclear translocation and HDAC activity supports pathway activation.	Activation of β-catenin signaling promotes UF cell proliferation, whereas inhibition of β-catenin or HDACs reduces proliferation markers, induces apoptosis, and causes cell-cycle arrest, thereby suppressing UF growth.	April 2020	[110]
Uterine fibroids	Matched MM and UF tissues	ChIP-seq of H3K27ac, promoter Capture Hi-C, RNA-seq, functional validation	AP-1 acts as a chromatin-associated regulator of enhancer activity	H3K27ac ChIP-seq data suggests enhancer dysfunction as a defining feature of UF transcriptional dysregulation	February 2020	[106]
Uterine fibroids	UF and MM tissues and primary cells	ChIP-seq, RNA-seq, ATAC-seq, Mutation analysis, KD or OE assay, IHC, DeCET method	Epigenetic changes in H3K27ac, H3K4me3, and H3K4me1 alter promoter and enhancer activity, mainly at distal enhancers forming large chromatin domains. These changes affect UF-related gene expression	These epigenetic alterations in UFs promote excess ECM and collagen deposition, dysregulate HOX developmental genes, and alter smooth-muscle cell phenotype and signaling pathways, including repression of the TGF-β signaling pathway.	March 2021	[105]
Uterine fibroids	Primary human UF tissues	DNA and RNA sequencing, ATAC-seq, ChIP-seq, and HiChIP to study chromatin interactions and epigenomic regulation in UF tissues.	Somatic and germline mutations in components of the SRCAP complex, including YEATS4 and ZNHIT1, impair deposition of the histone variant H2A.Z, altering the relationship between chromatin accessibility, DNA methylation, and transcriptional regulation.	Defective H2A.Z deposition leads to increased chromatin accessibility at transcription start sites, upregulation of gene expression, and activation of embryonic stem cell–like genes, resulting in epigenetic instability and aberrant cellular differentiation programs that contribute to UF tumorigenesis.	August 2021	[104]
Uterine fibroids	Human UF tissues and adjacent MM, and human UF primary cells collected from women undergoing myomectomy or hysterectomy	ELISA and gene expression analysis by q- PCR and Western blot; pharmacologic inhibition of HDACs; cell viability, proliferation markers, cell-cycle regulators, ECM proteins	Upregulation of HDACs increases histone deacetylation in UF cells, promoting transcription of genes involved in cell-cycle progression, ECM production, and TGFB3 signaling. HDAC inhibition by SAHA suppresses these transcriptional programs.	HDAC inhibition reduces UF cell proliferation, induces cell-cycle suppression, decreases ECM components (fibronectin and collagen I), and downregulates TGF-β3 and MMP9 expression, leading to inhibition of UF growth and matrix accumulation.	February 2022	[111]
Uterine fibroids	Human UF and matched MM tissues; primary UF cells treated with drug	Integrated RNA-seq (n = 48), H3K27ac ChIP-seq (*n* = 19), and qRT-PCR validation in SAHA-treated UF cells (*n* = 10); functional enrichment and differential gene expression analysis	Reduced H3K27ac levels in UFs compared with MM alters gene transcription, with hyperacetylation activating oncogenes and hypoacetylation repressing tumor-suppressor genes, affecting immune regulation, metabolism, and the TGF-β signaling pathway.	Dysregulation of cell proliferation, cell signaling, transport, angiogenesis, and ECM remodeling, contributing to UF development and maintenance	May 2022	[107]
Uterine fibroids	Human UF tissues and matched adjacent MM (RNA-seq *n* = 48; H3K4me3 ChIP-seq *n* = 19)	Integrated RNA-seq and H3K4me3 ChIP-seq analysis; differential histone methylation analysis; functional enrichment analysis	Epigenetic regulation via H3K4me3 affecting transcriptional regulation of oncogenes and tumor suppressor genes. Global suppression and gene-specific alterations of H3K4me3 modulate gene expression.	Dysregulated gene expression leading to aberrant cell proliferation, tumorigenesis, and activation of Wnt/β-catenin and TGF-β signaling pathways; altered neuronal/synapse-related gene programs; promotion of UF progression.	January 2023	[108]
Uterine fibroids	Human UF tissues and matched adjacent MM primary human UF cell cultures	RNA expression analysis, protein expression analysis, pharmacologic inhibition, adenoviral overexpression of EZH2, and chromatin immunoprecipitation (ChIP) assays	EZH2 regulates the DNA mismatch repair gene *MSH2* via H3K27me3. Increased EZH2 activity suppresses *MSH2* expression by promoting H3K27me3 enrichment at its promoter, while EZH2 inhibition reduces H3K27me3 levels and restores *MSH2* expression.	Dysregulation of *MSH2* expression contributes to altered DNA mismatch repair capacity in UFs, potentially promoting genomic instability and tumor development; *MSH2* may also serve as a potential biomarker for early detection of UFs	July 2023	[112]
Uterine fibroids	MMSCs and MM tissues from the Eker rat model exposed during development to the endocrine-disrupting chemical DES	Integrated epigenomic and transcriptomic analyses including RNA-seq, ChIP-seq, and RRBS, along with gain- and loss-of-function experiments, luciferase reporter assays, and pharmacologic inhibition of MLL1 and HDACs to assess regulation of estrogen-responsive and inflammatory genes.	Developmental exposure to DES epigenetically reprograms MMSCs via activation of MLL1 and DNA hypomethylation, leading to persistent activation of estrogen-responsive genes (ERGs) and inflammatory-responsive genes (IRGs). MLL1-dependent chromatin remodeling and HDAC-create a hyper-estrogenic and pro-inflammatory cellular state	Reprogrammed MMSCs display enhanced estrogen responsiveness and inflammatory signaling, and their secretome induces pro-inflammatory and immune-suppressive gene expression in neighboring myometrial cells via paracrine signaling, increasing susceptibility to hormone-dependent UF development later in life	July 2023, August 2023	[101,103]
Uterine fibroids	Human UF cells, and MM cells	EZH2 was modulated in UF cells via adenoviral overexpression or pharmacological EZH2 inhibition. qRT-PCR and immunoblot were used. Proliferation and apoptosis were evaluated.	EZH2 activates Wnt/β-catenin signaling by upregulating Wnt ligands, promoting β-catenin nuclear translocation and proliferation. Inhibition of EZH2, suppresses Wnt/β-catenin signaling, upregulates tumor suppressors and apoptotic markers, and selectively induces apoptosis in UF cells.	EZH2 overexpression promotes UF cell proliferation, while its inhibition suppresses proliferation and Wnt/β-catenin signaling. Methyl jasmonate selectively inhibits UF growth, reduces ECM proteins, induces apoptosis, and downregulates Wnt target genes, suggesting its potential as a nonhormonal therapy for UFs.	August 2023	[102]
Uterine fibroids	Human UF and matched MM, and primary UF cells treated with BRD9 inhibitors	Expression analysis of BRD9 in UF vs. MM; pharmacologic inhibition of BRD9; cell proliferation, apoptosis, and cell-cycle assays combined with high-throughput transcriptomic and bioinformatic pathway analysis	Upregulated BRD9 drives epigenetic transcriptional programs in UFs. BRD9 inhibition reprograms the epigenome and epitranscriptome, altering pathways related to cell-cycle progression, E2F targets, inflammatory response, ECM regulation, and m^6^A RNA modification	BRD9 inhibition induces apoptosis, causes cell-cycle arrest, suppresses cell proliferation, and reduces ECM deposition, thereby limiting UF growth and progression.	January 2024, January 2025	[113,114]
Uterine fibroids	Human UF and matched MM, and primary UF cells treated with inhibitors	Expression analysis of BET proteins in UF vs. MM; pharmacologic inhibition using BET inhibitors; cell viability and cell-cycle assays, plus transcriptomic profiling and bioinformatic pathway analysis	Dysregulated BET epigenetic regulators alter transcriptional programs controlling cell- cycle and signaling pathways. BET inhibition reprograms gene expression affecting multiple cellular pathways	BET inhibition reduces UF cell viability, induces cell-cycle arrest, and decreases ECM gene expression, suggesting suppression of UF growth and ECM accumulation.	December 2025	[115]

**Table 2:** The Role of Histone Modifications in Uterine Leiomyosarcoma.

Diseases	Sample Type	Approaches	Mechanism	Biological Effect	Publication Date	Ref.
uLMS	Human uterine tumor tissue samples, including UFs, HCLs, SMTs, LMSs, and ESTs	Immunohistochemical staining to assess expression of HDAC8 and compare it with established smooth muscle markers to evaluate diagnostic utility in differentiating uterine mesenchymal tumors	HDAC8 associated with smooth muscle differentiation, is selectively expressed in tumors with smooth muscle lineage, distinguishing them from stromal tumors that lack this differentiation.	HDAC8 expression is consistently detected in smooth muscle tumors and in areas of smooth muscle differentiation, while it is absent in conventional stromal tumor regions. This pattern supports its role as a specific marker of smooth muscle differentiation in uterine tumors.	March 2006	[132]
uLMS	Human LMS cell lines (SK-UT-1 and SK-LMS-1)	Retroviral FASN overexpression, siRNA KD, palmitate treatment, chromatin analysis including ChIP-seq, ChIP-PCR, and assessment of histone modifications	FASN drives the lipogenic phenotype of cancer and epigenomic reprogramming by altering histone modification enzymes, leading to changes in histone marks.	FASN overexpression enhances proliferation, migration, and cellular motility of uLMS cells. Epigenetic remodeling leads to chromatin changes and gene regulation (e.g., CRISP1 repression via H3K9me3), promoting a malignant phenotype.	June 2017	[130]
uLMS	Human tumor tissue samples from 42 patients analyzed using tissue microarray	IHC to evaluate expression of HDACs and p53, combined with analysis of histological subtype and survival outcomes	Dysregulated HDAC expression affecting epigenetic regulation in ULMS; interaction of HDAC5, HDAC7, and HDAC9 expression with p53 status and histological subtype in determining prognosis	HDACs show high and prevalent expression in tumors; lower expression of HDACs combined with p53 positivity or non-epithelioid subtype is associated with better disease-free survival; HDAC5 + epithelioid subtype identified as an independent predictor of poorer disease-free survival; HDACs may serve as prognostic biomarkers and potential therapeutic targets in uLMS.	August 2019	[133]
uLMS	uLMS cell lines, xenograft tumor models, and human uterine LMS and UF tissue samples analyzed by IHC	Evaluation of combined treatment with the tyrosine kinase inhibitor Pazopanib and hyperthermia; cell growth assays, xenograft tumor experiments, gene silencing of HAT1, promoter analysis of CLOCK, and IHC to assess expression levels and clinical correlations	Combined pazopanib and hyperthermia treatment suppresses the transcription factor CLOCK, leading to downregulation of HAT1. Reduced HAT1 levels decrease HAT1-mediated histone acetylation, thereby altering epigenetic regulation of gene expression in LMS cells.	Inhibition of HAT1 results in reduced uLMS cell proliferation and tumor growth, with the pazopanib–hyperthermia combination showing synergistic antitumor activity. High HAT1 expression is associated with more aggressive disease and poorer clinical outcomes in uLMS patients.	August 2020	[128]
uLMS	Human uLMS tumor and adjacent MM tissues; uLMS cell lines compared with benign UF and MM cell lines for functional and molecular analyses.	Expression analysis of BRD9 in tissues and cell lines; pharmacological inhibition of BRD9, cell proliferation, apoptosis, and cell-cycle assays; RNA-seq comparing; bioinformatics analyses for histone modifications and microRNA targets.	BRD9 is overexpressed in uLMS. Inhibition of BRD9 reprograms the oncogenic epigenome and alters transcriptional networks. BRD9 inhibition altered key pathways and also influences microRNA-mediated gene regulation and histone modification–associated gene sets.	Pharmacological inhibition of BRD9 suppresses uLMS cell proliferation, induces apoptosis, and causes cell-cycle arrest. Transcriptomic changes indicate disruption of oncogenic signaling networks and epigenetic regulatory programs, highlighting BRD9 as a therapeutic vulnerability and potential epigenetic target in uLMS.	July 2022	[134]
uLMS	Human uLMS tumor tissues, adjacent MM, and uterine tumor cell lines representing normal, benign, and malignant states	IHC and immunoblot analysis; pharmacological inhibition using Tucidinostat; gene expression profiling and gene set enrichment analysis (GSEA); epigenetic and transcriptomic analyses to examine changes in oncogenic pathways and microRNA–target interactions	Aberrant upregulation of Class I HDACs contributes to oncogenic epigenetic regulation in uLMS by promoting histone deacetylation and altering gene transcription. HDAC inhibition reprograms the oncogenic epigenome and modifies microRNA–gene regulatory networks.	Increased HDAC expression is associated with tumor progression from normal to malignant uterine cells, while HDAC inhibition suppresses uLMS cell proliferation and alters key oncogenic signaling pathways, suggesting therapeutic potential for HDAC-targeted treatments.	November 2022	[135]
uLMS	Human uLMS tumor tissues compared with MM tissues; functional studies performed in uLMS cellular models treated with BET inhibitors.	IHC used to evaluate expression of BET proteins; pharmacological inhibition using BET inhibitors; cell proliferation assays and cell-cycle analysis; RNA sequencing (RNA-seq) to identify pathway changes following BET inhibition.	BRD2-4 are overexpressed in uLMS and regulate transcriptional programs. Inhibition of BET proteins disrupts transcriptional regulation and alters multiple signaling pathways. BET inhibition also affects epigenetic regulatory networks.	BET protein inhibition suppresses uLMS cell proliferation and induces dose-dependent cell-cycle arrest. It also reprograms multiple oncogenic signaling and epigenetic pathways, suggesting that targeting BET proteins could be a potential epigenetic therapeutic strategy for uLMS.	August 2024	[116]
uLMS	LMS cell lines treated with HDAC inhibitors	Targeted inhibition of HDAC1, HDAC2, and HDAC3; analysis of TE and endogenous retrovirus expression; evaluation of interferon response signaling; investigation of A-to-I RNA editing mediated by ADAR; and assessment of histone modification levels	HDAC inhibition enhances chromatin accessibility and upregulates transposable elements, especially ERV1, producing dsRNAs that would typically trigger interferon signaling. However, elevated A-to-I editing by ADAR reduces dsRNA immunogenicity, dampening this response. Concurrently, HDAC inhibition increases H3K27ac at LTR12 regions, potentially activating pro-apoptotic genes.	HDAC inhibitors upregulate ERVs and transposable elements in LMS cells and modify chromatin activation marks. Despite increased dsRNA production, interferon signaling is not activated due to ADAR-mediated RNA editing. The epigenetic changes may enhance expression of pro-apoptotic genes, suggesting that combining HDAC inhibitors with ADAR inhibitors could promote tumor cell death and improve immunotherapy responses.	September 2024	[136]
uLMS	ULMS cell line (MES-SA) cultured in 2D monolayers and 3D matrigel-based spheroids	Treatment with EZH2 inhibitor (tazemetostat) and HDAC1/HDAC3 inhibitor (entinostat); cell proliferation, apoptosis, and cell- cycle assays; wound healing assay; RNA expression analysis, IHC; DNMT and HDAC activity measurements	EZH2 inhibition disrupts H3K27me3, reducing epigenetic silencing; HDAC inhibition by entinostat disrupts histone deacetylation and transcriptional repression; dual inhibition synergistically alters chromatin states, leading to cell-cycle arrest and apoptosis	Both single inhibitors suppressed cell proliferation, induced apoptosis, and caused cell-cycle arrest; entinostat additionally inhibited cell migration in 2D cultures; combination treatment enhanced all anti-tumor effects, demonstrating superior cytotoxicity against uterine sarcoma cells	June 2025	[131]
uLMS	Clinical tissues including uLMS tumors, normal MM, and UFs; human uLMS cell lines (SK-LMS-1 and SK-UT-1); and in vivo mouse xenograft tumors generated by subcutaneous implantation of uLMS cells in nude mice.	Real-time PCR and IHC; drug sensitivity assays using the SUV39H2 inhibitor OTS186935 and the PARP inhibitor olaparib; ChIP-seq	SUV39H2 inhibition regulates DNA double-strand break repair by recruiting γH2AX. Inhibition of SUV39H2 by OTS186935 reduces γH2AX accumulation and impairs DNA repair. When combined with the PARP inhibitor Olaparib, this defect produces a synthetic lethality effect, further compromising DNA repair pathways in uLMS cells.	SUV39H2 is overexpressed in uLMS compared with MM and UFs. OTS186935 reduces uLMS cell viability and inhibits double-strand DNA break repair. Combination therapy with OTS186935 and olaparib enhances antitumor activity and induces synthetic lethality, showing stronger tumor suppression both in vitro and in vivo.	December 2025	[129]
Metastatic LMS	Multicenter Phase II trial (NCT02303262); including a subset of uLMS patients	Oral mocetinostat at 70 mg three times weekly (escalated to 90 mg if tolerated) combined with intravenous gemcitabine at 1000 mg/m^2^ on days 5 and 12 of a 21-day cycle	HDAC inhibition with proposed reversal of gemcitabine resistance	The combination of mocetinostat and gemcitabine demonstrated modest clinical activity.	October 2018	[137]

**Table 3:** Targeting Histone Modification Regulators in Uterine Fibroids and Leiomyosarcoma.

Target	Inhibitors	Disease Context	Mechanism	Biological Effect	Publication Date	Ref.
HDACs	SAHA	UFs	Inhibit histone deacetylation	Upregulates the expression of tumor suppressor genes	May 2022	[107]
BRD9	TP-472, I-BRD9	UFs	Disrupts recognition of acetylated histones and BRD9-dependent transcriptional programs	Reduces proliferation, induces apoptosis, decreases ECM deposition; reprograms epigenome and epitranscriptome	January 2024, January 2025	[113,114]
EZH2	DZNep	UFs	Disrupts EZH2 activity	Epigenetic reprogramming of UF cells	October 2016	[112]
EZH2	Methyl jasmonate	UFs	Decreases EZH2 expression	Suppress cell proliferation	August 2023	[102]
BET proteins	JQ1, I-BET762	UFs	Inhibits binding to acetylated histones, suppressing oncogenic transcriptional programs	Promotes cell proliferation, survival, inflammatory signaling, and ECM accumulation.	December 2025	[115]
BET proteins	GS-626510	uLMS	Inhibits HDACs activity	Inhibits cell proliferation in uLMS PDX model	April 2021	[150]
BET proteins	JQ1, I-BET762	uLMS	Inhibits binding to acetylated histones, suppressing oncogenic transcriptional programs	Downregulates transcriptional addiction pathways and RNA modification regulators (e.g., FTO, YTHDC2, IGF2BP1)	August 2024	[116]
BRD9	TP-472	uLMS	Inhibits BRD9	Inhibits cell viability, and induces transcriptomic and epigenetic reprogramming	July 2022	[134]
EZH2	Tazemetostat	US	Inhibits H3K27me3, relieving transcriptional repression	Induce apoptosis and cell-cycle arrest	June 2025	[131]
HDACs	Tucidinostat, DL-sulforaph	uLMS	Inhibit HDAC activity	Reduces cell proliferation, and induce transcriptomic and epigenomic reprogramming	November 2022	[135]
HDACs	Entinostat	US	Block HDACs to increase histone acetylation, relaxes chromatin structure	Induce apoptosis and cell-cycle arrest, suppressed wound healing	June 2025	[131]
SUV39H2	OTS186935	uLMS	Blocks SUV39H2 activity	Decreases cell viability, and impairs DNA damage repair, and loss of H3K9me3-mediated chromatin repression. These effects sensitize tumor cells to PARP inhibition, resulting in synthetic lethality and enhanced antitumor efficacy both in vitro and in vivo.	December 2025	[129]

## Data Availability

No new data were created or analyzed in this study. Data sharing is not applicable.

## Abbreviations

The following abbreviations are used in this manuscript:

ADP-RH	ADP-ribosylhydrolases
ATAC-seq	Assay for Transposase-Accessible Chromatin sequencing
BET	Bromodomain and Extra-Terminal domain
BRCA1	Breast Cancer Gene 1
BRD2/3/4/9	Bromodomain-containing proteins 2/3/4/9
ChIP-PCR	Chromatin Immunoprecipitation PCR
ChIP-seq	Chromatin Immunoprecipitation sequencing
CLOCK	Circadian Locomotor Output Cycles Kaput
CRISP1	Cysteine Rich Secretory Protein 1
CTGF	Connective Tissue Growth Factor
DeCET	Deconvolution-based Chromatin Epigenetic Tool
DES	Diethylstilbestrol
DNMT	DNA Methyltransferase
DUBs	Deubiquitinases
DZNep	3-Deazaneplanocin A
ECM	Extracellular Matrix
EGCG	Epigallocatechin-3-gallate
ELISA	Enzyme-Linked Immunosorbent Assay
ELT-3	Eker Leiomyoma Tumor Cell Line
EMT	Epithelial–Mesenchymal Transition
ERα/ESR1	Estrogen Receptor Alpha
ERG	Estrogen-Responsive Gene
ERV	Endogenous Retrovirus
ERV1	Endogenous Retrovirus 1
EZH2	Enhancer of Zeste Homolog 2
FASN	Fatty Acid Synthase
GSEA	Gene Set Enrichment Analysis
H2A.Z	Histone Variant H2A.Z
H2AK119ub	Histone H2A Lysine 119 Ubiquitination
H2BK120ub	Histone H2B Lysine 120 Ubiquitination
H3K27ac	Histone H3 Lysine 27 Acetylation
H3K27me3	Histone H3 Lysine 27 Trimethylation
H3K4me1	Histone H3 Lysine 4 Monomethylation
H3K4me3	Histone H3 Lysine 4 Trimethylation
H3K9me3	Histone H3 Lysine 9 Trimethylation
HAT	Histone Acetyltransferase
HAT1	Histone Acetyltransferase 1
HCL	Highly Cellular Leiomyoma
HDAC	Histone Deacetylase
HDACI	Histone Deacetylase Inhibitor
HDM	Histone Demethylase
HiChIP	High-throughput Chromatin Conformation Capture with Immunoprecipitation
HMGA2	High Mobility Group AT-hook 2
HOXA13	Homeobox A13
I-BET762/JQ1	BET Inhibitors
I-BRD9/TP-472	BRD9 Inhibitors
IHC	Immunohistochemistry
IRG	Inflammatory-Responsive Gene
KRAS	Kirsten Rat Sarcoma Viral Oncogene
KD	Knockdown
LMS	Leiomyosarcoma
LTR12	Long Terminal Repeat 12
MED12	Mediator Complex Subunit 12
MJ	Methyl Jasmonate
MMP9	Matrix Metallopeptidase 9
MM	Myometrium
MMSC	Myometrial Stem Cell
mTORC1	Mechanistic Target of Rapamycin Complex 1
MYC/c-Myc	MYC Proto-Oncogene
NF-κB	Nuclear Factor Kappa B
OE	Overexpression
PARP	Poly (ADP-ribose) Polymerase
PTMs	Post-Translational Modifications
qRT-PCR	Quantitative Real-Time Polymerase Chain Reaction
RAD51	RAD51 Recombinase
RNA-seq	RNA Sequencing
RRBS	Reduced Representation Bisulfite Sequencing
SAHA	Suberoylanilide Hydroxamic Acid
siRNA	Small Interfering RNA
SMT	Smooth Muscle Tumor
SRCAP	SNF2-related CREBBP Activator Protein
SUV39H2	Suppressor of Variegation 3–9 Homolog 2
TE	Transposable Element
TGF-β/TGFB3	Transforming Growth Factor Beta/Isoform 3
UF	Uterine Fibroid
uLMS	Uterine Leiomyosarcoma
US	Uterine Sarcoma
USMTs	Uterine Smooth Muscle Tumors
VitD	Vitamin D
YEATS4	YEATS Domain Containing 4
ZNHIT1	Zinc Finger HIT-Type Containing 1
γH2AX	Phosphorylated H2A Histone Family Member X
